# The Ageing Brain: Investigating the Role of Age in Changes to the Human Cerebral Microvasculature With an *in silico* Model

**DOI:** 10.3389/fnagi.2021.632521

**Published:** 2021-08-05

**Authors:** Barnaby J. Graff, Stephen J. Payne, Wahbi K. El-Bouri

**Affiliations:** ^1^Department of Engineering Science, Institute of Biomedical Engineering, University of Oxford, Oxford, United Kingdom; ^2^Liverpool Centre for Cardiovascular Science, University of Liverpool & Liverpool Heart and Chest Hospital, Liverpool, United Kingdom; ^3^Department of Cardiovascular and Metabolic Medicine, Institute of Life Course and Medical Sciences, University of Liverpool, Liverpool, United Kingdom

**Keywords:** capillaries, cerebral blood flow, healthy ageing, micro-emboli, neurodegenaration

## Abstract

Ageing causes extensive structural changes to the human cerebral microvasculature, which have a significant effect on capillary bed perfusion and oxygen transport. Current models of brain capillary networks in the literature focus on healthy adult brains and do not capture the effects of ageing, which is critical when studying neurodegenerative diseases. This study builds upon a statistically accurate model of the human cerebral microvasculature based on *ex-vivo* morphological data. This model is adapted for “healthy” ageing using *in-vivo* measurements from mice at three distinct age groups—young, middle-aged, and old. From this new model, blood and molecular exchange parameters are calculated such as permeability and surface-area-to-volume ratio, and compared across the three age groups. The ability to alter the model vessel-by-vessel is used to create a continuous gradient of ageing. It was found that surface-area-to-volume ratio reduced in old age by 6% and permeability by 24% from middle-age to old age, and variability within the networks also increased with age. The ageing gradient indicated a threshold in the ageing process around 75 years old, after which small changes have an amplified effect on blood flow properties. This gradient enables comparison of studies measuring cerebral properties at discrete points in time. The response of middle aged and old aged capillary beds to micro-emboli showed a lower robustness of the old age capillary bed to vessel occlusion. As the brain ages, there is thus increased vulnerability of the microvasculature—with a “tipping point” beyond which further remodeling of the microvasculature has exaggerated effects on the brain. When developing *in-silico* models of the brain, age is a very important consideration to accurately assess risk factors for cognitive decline and isolate early biomarkers of microvascular health.

## 1. Introduction

The human brain is the central organ of the nervous system, accounting for 2% of the body's mass but 14% of the volumetric blood flow (Fantini et al., 2016). The cerebral microvasculature is the primary surface for perfusion and oxygen transport in the brain and is therefore crucial to maintaining functionality and quality of life. Ageing is associated with deterioration of this capacity and in abnormal cases the onset of neurodegenerative diseases.

The importance of the impact of ageing is brought into focus by the increasingly ageing global demographic. In the UK, the number of people over the age of 65 is expected to reach 26% of the population by 2066 (Office for National Statistics, 2018). Many of these individuals will suffer from neurological disorders caused by extrinsic factors such as viral or bacterial infections (e.g., multiple sclerosis or meningitis, respectively), or which develop intrinsically either due to acute events (traumatic brain injury) or chronic illness [small vessel disease (SVD), dementia] (Rosenberg, 2014). Dementia is now the leading cause of death in the UK (John, 2019). Age is the common denominator in the majority of these cases, with approximately 32% of people over the age of 85 affected (Hebert et al., 2013). There is now thought to be a large overlap between vascular dementia and AD, with most patients having a form of “mixed dementia” centered on the permeability of the blood-brain barrier (Javanshiri et al., 2018). Therefore it is critical to understand the ageing process of the human cerebral vasculature.

Significant changes to brain anatomy are seen with increasing age. A decrease in overall volume by 5% per decade after age 60 (Hedman et al., 2012) and reduced angiogenesis leading to reduction in vessel density (Riddle et al., 2003; Murugesan et al., 2012) are common even in “healthy” ageing. Increasing vessel stiffness, tortuosity and hypoperfusion (Xu et al., 2017) also develop, even without confounding pathological factors. These statistics vary widely between individuals, population demographics and individual regions of the brain. Assessing “normality” in terms of the ageing brain is thus a difficult and imprecise process. However, brain performance is ultimately judged on functionality which affects everyday quality of life, such as memory and processing speed. There is still a lack of understanding in linking measurable brain characteristics to cognitive function in the face of age-related performance decline (Glisky, 2019). The cerebral microvasculature is increasingly being seen as key to understanding global level perfusion and oxygen transport (Lauwers et al., 2008) and therefore a key area of research interest.

In spite of this, measuring changes in the microvasculature *in-vivo* is still a significant challenge. This is due to the imaging resolution of industry-standard technologies such as Magnetic Resonance Imaging (MRI), Positron Emission Tomography (PET) and Perfusion Computed Tomography (PCT) being unable to detect vessels with diameter of less than c.1mm (Wintermark et al., 2005). The average diameter of human capillaries is around 6μm (Cassot et al., 2006). Models have previously been developed to simulate the cerebral microvasculature in rodents (Reichold et al., 2009; Blinder et al., 2010; Schmid et al., 2017) and in humans (Lorthois et al., 2011; Linninger et al., 2013; El-Bouri and Payne, 2015). However, much of the research into computational modelling of the brain has focused on understanding the mechanisms of a healthy young adult at a discrete point in time.

As such, in this study we develop a model of ageing human brains based on El-Bouri and Payne (2015) which creates statistically accurate networks of old-age capillaries. These periodic structures can be homogenised to derive macro-scale flow equations for the capillary bed. Permeability and molecular exchange parameters are then calculated to assess how ageing alters perfusion and blood flow. A continuous gradient of ageing is also created to overcome the difficulties of discrete datasets in the literature, and to attempt to chart the shape of “healthy” decline. Vulnerability of the brain due to ageing is then assessed by simulating micro-emboli and comparing the response of a healthy adult brain and a healthy old-aged brain.

## 2. Materials and Methods

### 2.1. Inter-species Vascular Morphology

The cerebral capillary bed model developed in Su et al. (2012) and extended in El-Bouri and Payne (2015) forms the starting point for this research. This model has also previously been used to scale-up the capillary bed to model blood flow in large regions of the brain computationally efficiently (El-Bouri and Payne, 2018). The model represents the cerebral capillary bed of a healthy adult, with data extracted from the collateral sulcus in the temporal lobe of a preserved brain (Duvernoy et al., 1981).

A recent study uses two-photon phosphorescence microscopy to measure the cerebral properties of mice microvasculature *in-vivo* (Moeini et al., 2018). Mice have long been used as a basis for biomedical research, due to similar genetic and structural properties to humans. Previous work suggests that cerebral capillary networks between the two species are topologically equivalent, and that geometric metrics differ only in scaling (Smith et al., 2019b). Therefore data from mice are used here as the basis for updating our existing model of the human microvasculature to represent the ageing process.

**Figure 1:**
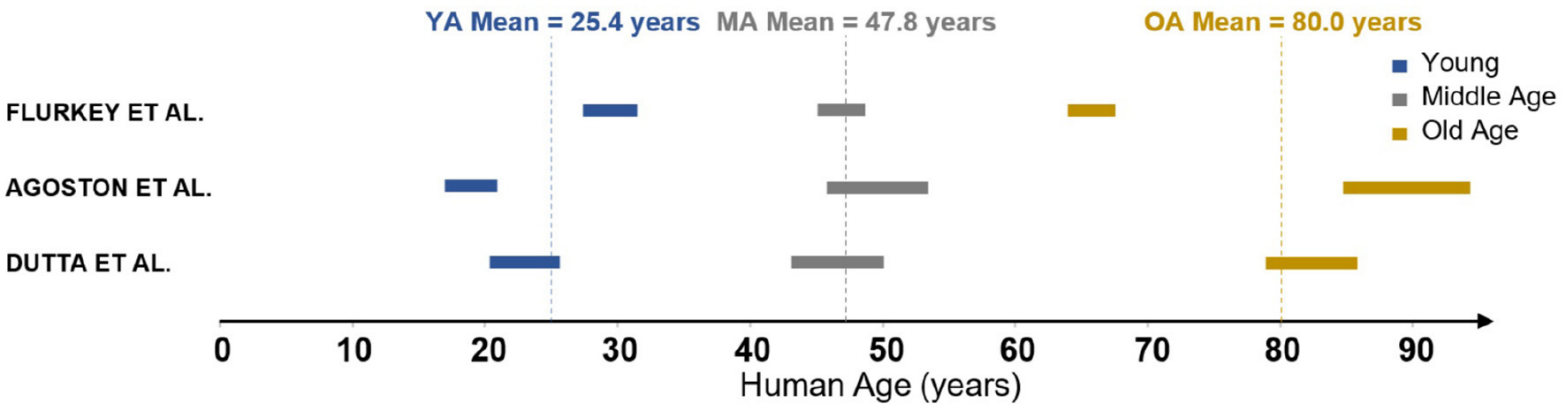
Mouse-human age conversion identified in the literature.

The measurements in Moeini et al. (2018) were performed on three groups of C57BL/6J male mice of age 6-8, 13–15, and 24–26 months old, respectively. These groups are characterised as young (YA), middle-aged (MA) and old (OA). In order to understand these ages relative to human life expectancy, three reviews of mouse-to-human age conversion were identified (Flurkey et al., 2007; Dutta and Sengupta, 2016; Agoston, 2017). These reviews discuss the relative length of different periods of life, from childhood through puberty all the way through senescence. The age group results of 6–8, 13–15, and 24–26 months converted to human years following the methodology in the literature are summarised in Figure 1. There is clearly a range of values in the literature rather than a widely accepted conversion framework. The biggest discrepancy is in the parameterisation of an “old” mouse, as two of these reviews suggest that at 24 months there are commonly signs of dementia and severe cerebrovascular degradation present. As the mice in Moeini et al. (2018) are extensively imaged and shown to be healthy, the mean age of the old group is considered to be an overestimation. Therefore YA, MA, and OA were chosen to correspond to 25, 45, and 75 years old, respectively, although the methodology presented here could easily be adjusted to any changes in these values.

### 2.2. Ageing Brain Model Development

Data measured in mice are used to adapt the human brain model from El-Bouri and Payne (2015) to model the ageing process. The human cerebral capillary bed is represented by voxels of 375μm side length containing statistically accurate microvascular networks randomly generated through the minimum-spanning tree method (Su et al., 2012). A length scale of 375 μm was used as this was found to be the representative elementary volume large enough to be characteristic of the large-scale capillary network. The networks match many geometric properties of the capillary network such as vessel length distributions, diameter distributions, and connectivities of the network measured from studies analysing India-ink injected brain regions using laser microscopy (Cassot et al., 2006). A set of 498 statistically accurate microvascular networks from El-Bouri and Payne (2015) was taken and split into three groups. One of these groups of 166 cubes was left unmodified to represent the middle-aged set of networks, as the original model represents the microvasculature of a healthy middle-aged adult brain. The other two sets were altered according to the ageing algorithm described below to model young and old brain microvasculatures.

The need to characterise a scaling factor between mice and humans for every geometrical property is eliminated by using a ratio method, comparing young and old networks to the middle-aged baseline. The data available in Moeini et al. (2018) and subsequently used in this work describe changes of vessel radius, haematocrit, resistance and density with ageing. These scalings are then taken and used to generate separate young and old networks via the ageing algorithm proposed here:

1. The radius is scaled in the young and old groups;
2. The haematocrit is scaled in the young and old groups;
3. A new viscosity is calculated for the vessels in the networks assuming haematocrit is constant in each individual network at the representative value for the age group (Pries et al., 1992);
4. A new conductance for each vessel can be calculated using Poiseuille's law where the conductance is $\frac{\pi r^{4}}{8\mu L}$, *r* is the radius, μ the viscosity, and *L* the length of the vessel;

Table 1 shows the ratios of key parameters from Moeini et al. (2018) used to scale the networks.

**Table 1:** Parameters used at each age group to scale the capillary networks—ratios obtained from Moeini et al. (2018).

	**YA ratios**	**MA ratios**	**OA ratios**
Radius	1.00	1.00	1.07
Haematocrit	1.04	1.00	0.81
Volume density	0.91	1.00	0.79
Cerebral blood flow	0.71	1.00	0.79

At this point, an assumption is made that the length of individual vessels remains constant throughout the ageing process. This is consistent with the literature as the cerebral microvasculature is shown not to spatially remodel with ageing, and although a mild increase in tortuosity is expected (Bullitt et al., 2010) this has a minimal effect on vessel resistance and flow (Han, 2012).

Two different methods were next implemented to calculate the expected change in vessel number density *n* with ageing (number of vessels per unit volume). The first method randomly prunes vessels from the network structure to match the volumetric density change from the baseline middle-age group measured in Moeini et al. (2018). As individual vessels are randomly pruned from the network, the volume density of vessels remaining is calculated. When this value matches the expected volume density reduction compared to middle-age, the corresponding number density reduction can be calculated using the absolute number of vessels pruned to match a specific volume reduction.

The second method randomly prunes vessels from the network structure to match the cerebral blood flow (CBF) change from the baseline middle-age group measured in Moeini et al. (2018). By removing a single vessel at a time, the model can be finely tuned to the ageing parameters, with the number density reduction calculated for each individual cube and averaged over the age group set. For continuity, all of the blood flowing into a region must also flow out. Therefore periodic distribution of the surface nodes is maintained on each unit volume for homogenisation purposes. This allows the model to represent large volumes of the cerebral capillary bed through a homogenised permeability tensor.

### 2.3. Key Model Parameter Calculations

The model allows a homogenised 3-D permeability tensor to be calculated for the vascular networks. This links the microvasculature to macro-scale blood flow properties to enable the development of continuum models of the cerebral capillary bed. By defining the ease with which blood can flow through a particular region, the effect of ageing can thus be quantified in terms of flow and molecular exchange parameters.

**Figure 2:**
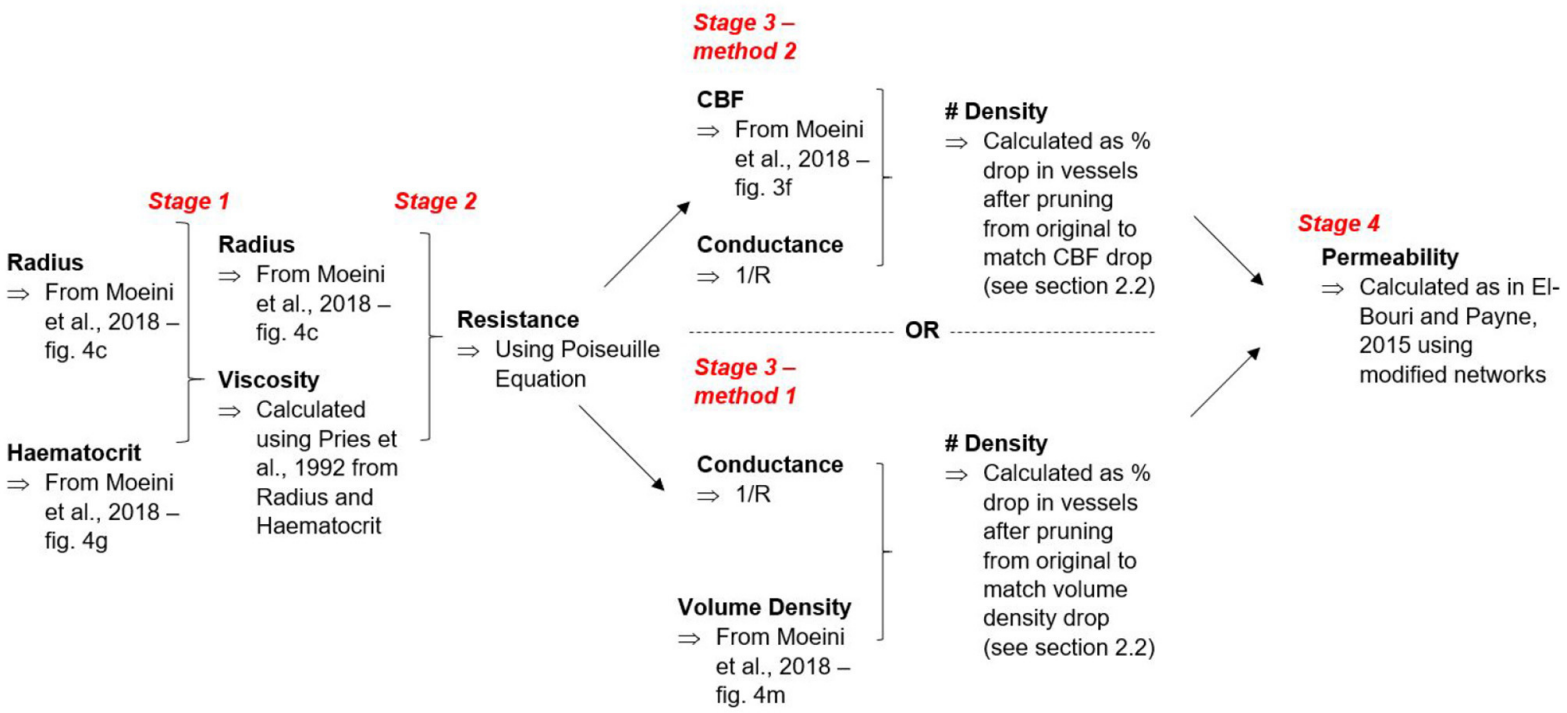
Flowchart mapping route of calculations.

As previously described in El-Bouri and Payne (2015), the permeability is calculated by solving a periodic boundary value problem over the generated cubes of the capillary network. The Poiseuille equation is used to find the flux through each individual capillary vessel. Pressure gradients are then imposed on each principal direction to create a 3 × 3 tensor. The permeability tensor **K** is calculated in terms of the resolved flux through cube surfaces, cube surface area, and pressure gradients.

(1) $$K_{ij}=\frac{\sum q_{m,surf}^{j}}{\Gamma^{j}\nabla p^{j}}i,\;j=1,2,3$$

where ${\text{q}}_{m,surf}^{j}$ is surface outflow in j^*th*^ direction, Γ^*j*^ is outflow surface area and *p*^*j*^ is the pressure gradient in the j^*th*^ direction. In this case, *i* and *j* refer to Cartesian co-ordinates. The flowchart in Figure 2 summarises both of the methods for calculating vessel number density and how these outputs are taken to calculate permeability.

Permeability is a robust proxy for understanding perfusion, but further parameters are required to understand molecular exchange. The capillaries are the primary exchange medium for molecules within the brain (Hadjistassou et al., 2015)—albeit the penetrating arterioles also play a key role in oxygen transport to tissue (Sakadžić et al., 2014). Surface-area-to-volume ratio (SA:Vol) and volumetric density give an indication of how effectively they continue to deliver these molecules in spite of ageing. SA:Vol is important because the large surface area of the vessels in the cerebral compartment drives molecular exchange in the vasculature. As with permeability, SA:Vol and density are variables derived from the homogenisation of the equations describing blood flow and molecular exchange on the local scale (Shipley and Chapman, 2010).

### 2.4. Interpolating Between Discrete Age Groups

A limitation of the data presented in Moeini et al. (2018) is the discrete nature of the age group measurements. Brain ageing varies greatly between individuals due to genetic factors such as *APOE* ϵ*4* (Pietzuch et al., 2019) and demographic differences such as sex and education (Curiati et al., 2009). “Healthy” ageing is hard to define as common pathologies such as hypertension and diabetes have a significant impact on the way the brain ages (Gasecki et al., 2013). In addition to these medical effects on the cerebral microvasculature, there is also evidence that lifestyle differences such as diet, education level and exercise can have a marked effect (Ungvari et al., 2018; Walters et al., 2018). These are not considered here but will be an important topic for future research. Many human studies measure discrete intervals in time on small, precise cohorts which makes comparison between papers difficult. The model that we propose here, however, allows assessment of how the brain deteriorates “healthily” over time by proposing a continuous pathway using interpolation between ages. The ageing process from middle-age to old-age was focused on as this is the time frame within which most age-associated neurological diseases manifest.

From a literature review of regional cerebral blood flow (CBF) changes with ageing, an expected drop per year of 0.42% ± 0.08 was found—a typical value for association regions responsible for complex processing tasks (Chen et al., 2011; Aanerud et al., 2012; De Vis et al., 2015; Leoni et al., 2017; Jezzard et al., 2018). In this paper we use the average of 0.42%. The cube flow reduction caused by pruning one vessel at a time was found, taking an average over all 166 cubes in each set. It was assumed, consistent with findings in this work, that CBF and permeability drop at the same rate (i.e., driving pressure remains the same with ageing). In fact, the computed permeability can be used as a robust surrogate for CBF when considering small volumes of tissue. It is important to note that this model considers normotensive ageing, but hypertension and other comorbidities are very common in the ageing population. This permeability drop per pruned vessel was then compared to the expected permeability drop (or effectively CBF drop) per year. This allowed a characterisation to be made of the number of vessels being naturally removed per year.

Physical characteristics (vessel radius and conductance) are accounted for here in the ageing gradient by linearly interpolating between the measurements in middle-age (MA) and old-age (OA). The groups that we used here are MA, OA 50 (physical characteristics half way between MA and OA), OA 75 (physical characteristics 75% of way from MA to OA) and OA. These fractions are chosen to capture the point at which changing physical characteristics begins to have an influence on permeability drop with ageing. No difference in gradient of permeability decline is seen with smaller changes. The statistics used for each set are summarised in Table 2.

**Table 2:** Physical characteristic ratios for interpolation between age groups.

	**MA**	**OA 50**	**OA 75**	**OA**
Radius	1.00	1.03	1.05	1.07
Conductance	1.00	1.16	1.24	1.32

These statistics were used to generate four different rates of ageing to represent decades from age 45 to 85, which could be combined to provide an overall graded chart. By comparing percentage vessel decline against number of years and percentage vessel decline against permeability drop, a decline in permeability per year in association regions of the brain can be calculated in four 10-year phases. The permeability is matched at the end of each 10 year period and the physical characteristics of the network changed to simulate ageing, starting from a MA brain at age 45. This gives a chart interpolating between ages, starting from a healthy, middle-aged adult brain.

### 2.5. Simulating Micro-Emboli in the Ageing Brain

The ageing process leads to substantial changes in the cerebral microvasculature which are next incorporated into a 3-D model through methodology described in the previous sections. This model can be used to test how the brain capillary bed responds to events differently through a human lifetime. In this section, the effect of micro-emboli on the brain is investigated. These micro-infarcts occur at a scale where *in-vivo* detection is challenging and therefore computer simulation is important in assessing these effects. It has previously been shown *in-silico* that the severity of micro-strokes in mice is strongly linked to vascular topology (Schmid et al., 2021) and therefore this simulation quantifies increased vulnerability to micro-stroke introduced through the natural ageing process.

Computationally, a micro-embolism follows the same process as pruning a vessel. A no-flow condition is imposed on an individual capillary to simulate a clot fragment preventing blood flow. For 166 middle-age cubes and 166 old-age cubes, vessels are blocked randomly, one-by-one until the cube permeability reaches zero. The fractions of vessel number, vessel volume and vessel surface-area blocked in the networks are recorded for each micro-emboli and plotted against the permeability fraction. A permeability reduction rate can therefore be calculated as (% permeability reduction / % blockage fraction).

## 3. Results

### 3.1. Quantification of Separate Ageing Effects

**Figure 3:**
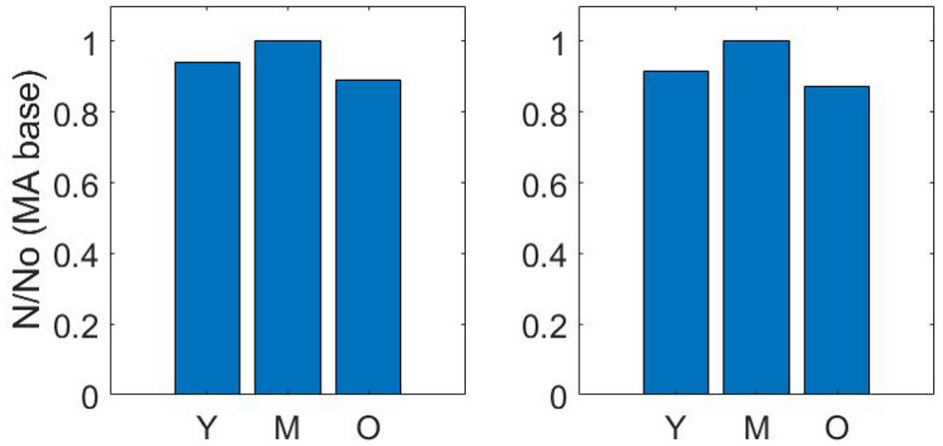
Number density ratios using volume density matching **(left)** and cerebral blood flow matching **(right)**—Y = Young; M, Middle-age; O, Old-age.

Pruning vessels by matching cerebral blood flow (described in section 2.2) gives a similar reduction in number density to pruning vessels by matching the volume density from Moeini et al. (2018). The number density ratios for the three age group sets of young, middle-aged and old, where the baseline middle-age is 1.00 are 0.92 : 1.00 : 0.87 and 0.94 : 1.00 : 0.89 for matching of CBF and volume density, respectively (Figure 3).

**Figure 4:**
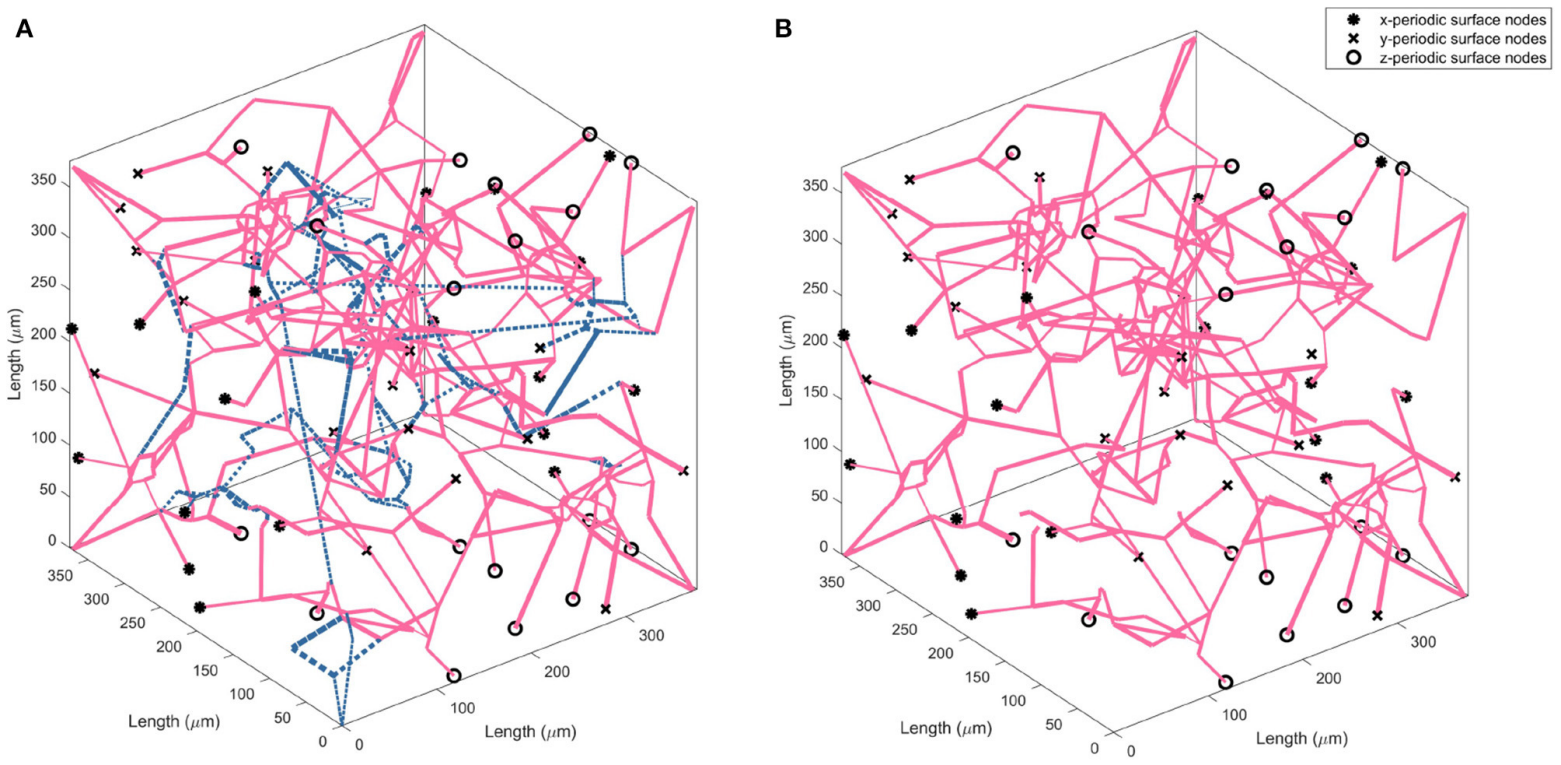
Visualising the change in vessel parameters in a cubic volume of the ageing brain with a middle-age network **(A)** and old-age network **(B)**. Blue dashed lines indicate the vessels pruned to achieve an accurate old-age vessel density.

The drop calculated using both methods is within the expected 10–20% range (Leenders et al., 1990; Desjardins et al., 2014; Nagata et al., 2016). Due to the equivalence in results of both methods, a decision was made to continue with just one method for the remaining calculations in the paper. Therefore, the flow matching method is used to calculate all future permeability. Figure 4 illustrates an example of the CBF pruning algorithm. The network structure before and after pruning 13% of vessels is shown—the expected number density reduction moving from middle-age to old-age.

**Figure 5:**
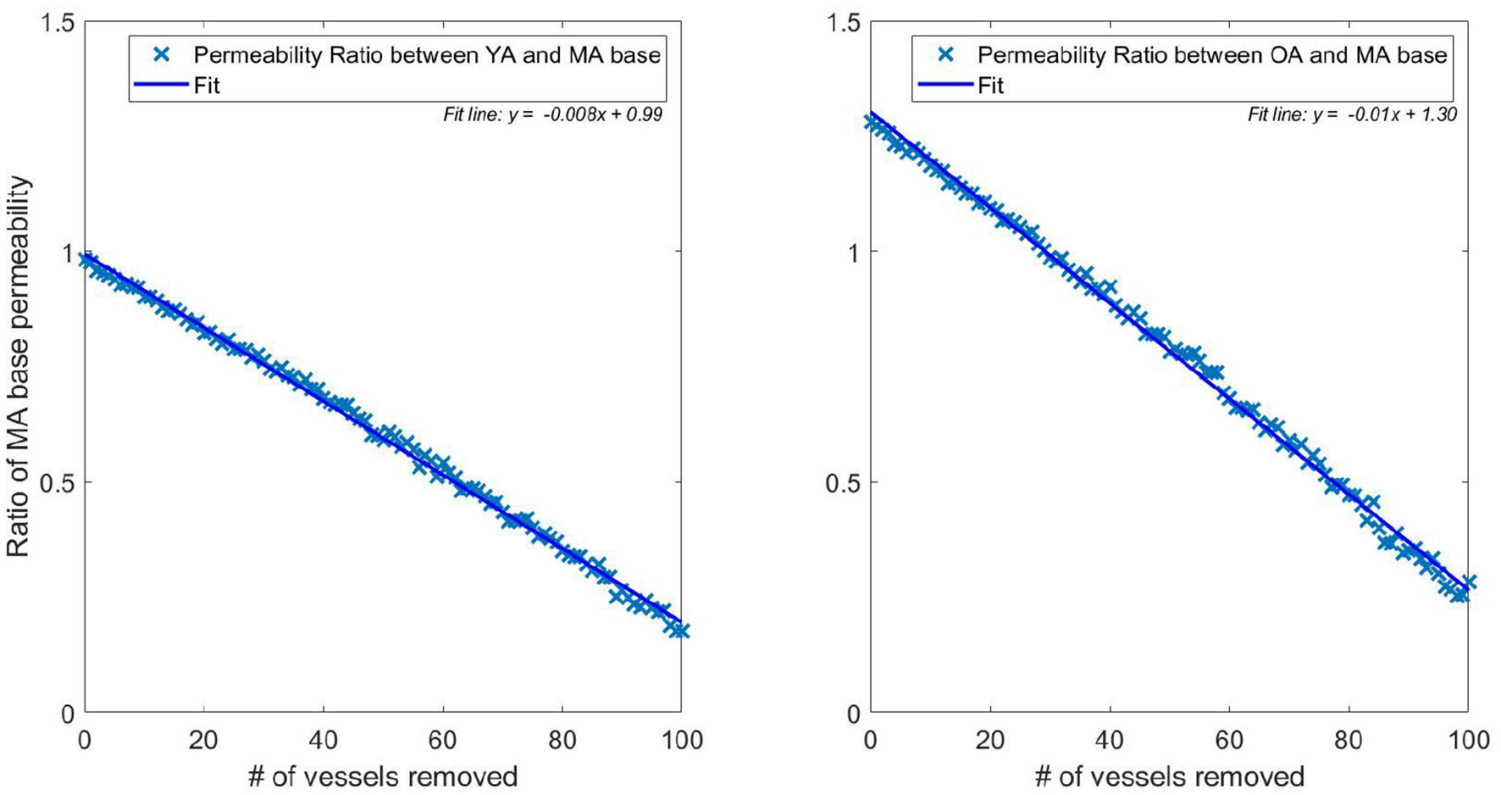
Ratio of pruned network permeability to the unpruned MA base permeability (data points represent the average permeability over 166 networks for each specific number of pruned vessels). Note that the YA, MA, and OA brains all start with the same vessel density—only radius and viscosity is modified for the different age groups. This elucidates the impact of removing individual vessels on permeability in young **(left)** and old **(right)** brains.

The flexibility of the model allows isolation of the two separate quantities influencing the drop in flow (and therefore permeability) with age. Figure 5 plots the permeability ratios of young and old networks against the MA base permeability, to compare how the physical property changes (radius dilation, conductance) affect the network before and after vessels are removed (each data point represents the average over 166 networks). When no vessels are removed, this is simulating the effect of no reduction in vessel density vs. MA baseline whilst continuing to model the physical changes of ageing. Although not a physiologically accurate process, this illustrates the flexibility of the capillary network in responding to loss of vessel density. It is therefore possible to understand how dilation contributes on its own to capillary network robustness in ageing. Figure 5 shows that the conditions of increasing diameter and decreasing viscosity would actually increase the permeability to 1.3 times the middle aged value if the same vessel density as in the MA group remained. The percentage reduction in permeability (shown in Figure 5) and flow per vessel are equal for each age group, and see a 0.8% drop per vessel removed in the young group and a 1.0% drop per vessel in the old group.

This illustrates a potential mechanism by which the brain partly accounts for the reduction in vessel density. The dilation of vessels allows a greater volumetric flow, increasing conductance and therefore perfusion from the remaining vessels. The flow in the YA and OA sets was matched to 71 and 79% of the MA flow, respectively, in Moeini et al. (2018), but flow through individual vessels increases in old age compared to middle age due to dilation of the remaining vessels. This physiological reaction to the decrease in vessel density could potentially be due to the autoregulation mechanisms in the brain. In younger subjects, vessel vasoconstriction can protect against development of hypoxic regions but this capacity reduces due to the stiffening and dilating of capillaries with age (Wagner et al., 2012).

### 3.2. Capillary Network Permeability

**Figure 6:**
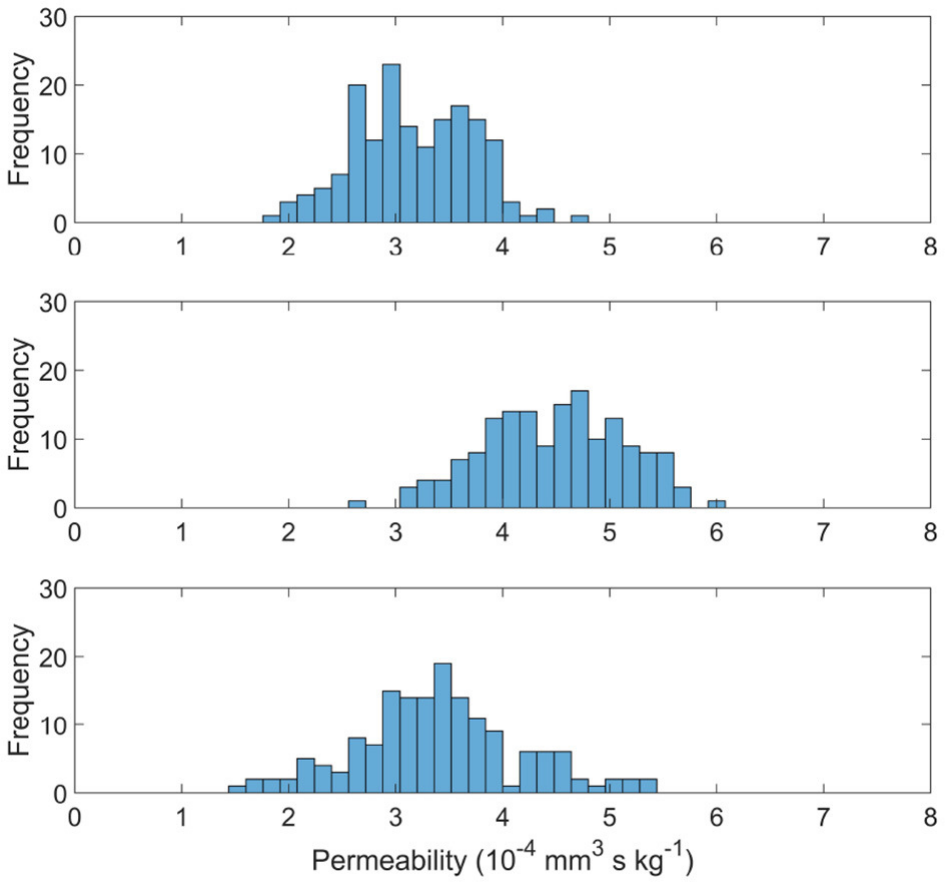
Permeability distributions for young **(top)**, middle aged **(middle)**, and old **(bottom)** capillary networks.

Figure 6 shows the distribution of permeability values in the measured cubes of each age set. The permeability tensor was found to be isotropic with off-diagonals distributed around 0, as in El-Bouri and Payne (2015). The off-diagonal permeability values were on average two orders of magnitude smaller than the permeability of the principal terms. Therefore simply the first term of the tensor **K** is presented in this paper. The mean and variance of the permeability values are (3.17 ± 0.55) × 10^−4^ mm^3^ s kg^−1^, (4.48 ± 0.66) × 10^−4^ mm^3^ s kg^−1^ and (3.39 ± 0.77) × 10^−4^ mm^3^ s kg^−1^ for YA, MA, and OA sets, respectively. Interestingly, the permeability drops in both YA and OA compared to the middle-aged set. CBF would normally be expected to be largest in the young population and therefore so would permeability (Prince et al., 2013). However, CBF is used as an input for the model when pruning vessels (Figure 2) and CBF input for the young age is lower than the MA in mice, leading to this “inverted U shape” trend. There is also shown to be an increase in variability with ageing through the three age group sets.

### 3.3. Molecular Exchange Parameters

**Figure 7:**
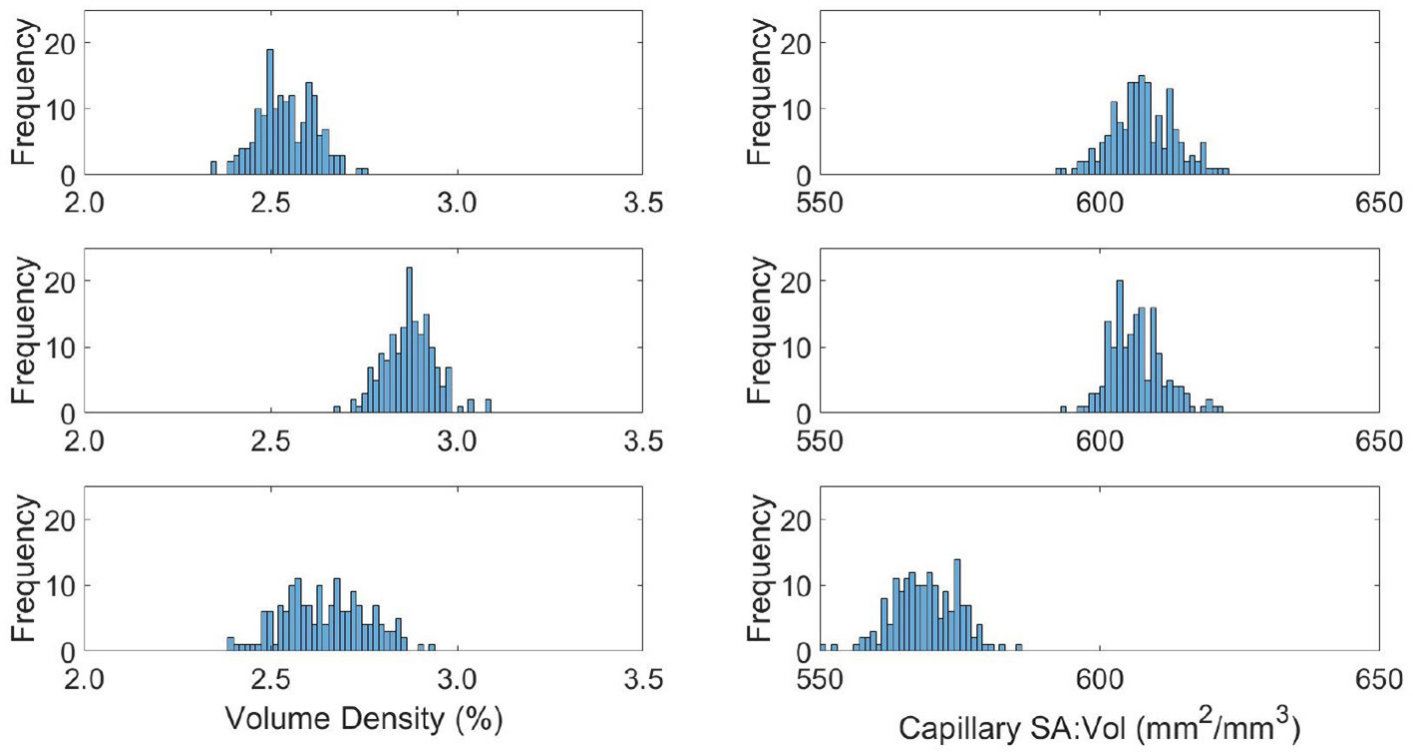
Distributions of capillary volume fraction **(left)** and surface-area-to-volume ratio of the capillaries **(right)** in young **(top)**, middle aged **(middle)**, and old **(bottom)** capillary networks.

The capillary density and surface-area-to volume ratio (SA:Vol) distributions are shown in Figure 7, with the mean and variance statistics summarised in Table 3.

**Table 3:** Quantified mean and variation of model oxygen parameters.

	**YA**	**MA**	**OA**
Density (%)	2.5 ± 0.08	2.9 ± 0.07	2.6 ± 0.11
SA:Vol ($\frac{mm^{2}}{mm^{3}}$)	607 ± 5	608 ± 6	569 ± 6

The relative results of YA, MA and OA groups between volume density and SA:Vol show different patterns of development with ageing. The volume density in YA and OA groups is similar, which matches the overall shape of permeability histograms in Figure 6. However in the OA group, SA:Vol is considerably reduced relative to MA and YA. This suggests that the capacity for molecular exchange into the tissue is substantially reduced in old age.

A likely cause is the increase in average capillary radius in the OA set. Due to rarefaction of the network, capillaries dilate to maintain the required perfusion through a specific tissue volume. Comparably in dementia patients, chronic hypoperfusion causes capillary dilation and cerebral microvessel restructuring that affects brain function (Hase et al., 2019). A future aim is to use these data with an oxygen transport model previously developed in the research group (El-Bouri et al., 2019). Understanding the potential development of hypoxic regions due to ageing will show to what extent this is normal in “healthy” brains.

### 3.4. Continuous Ageing Gradient

**Figure 8:**
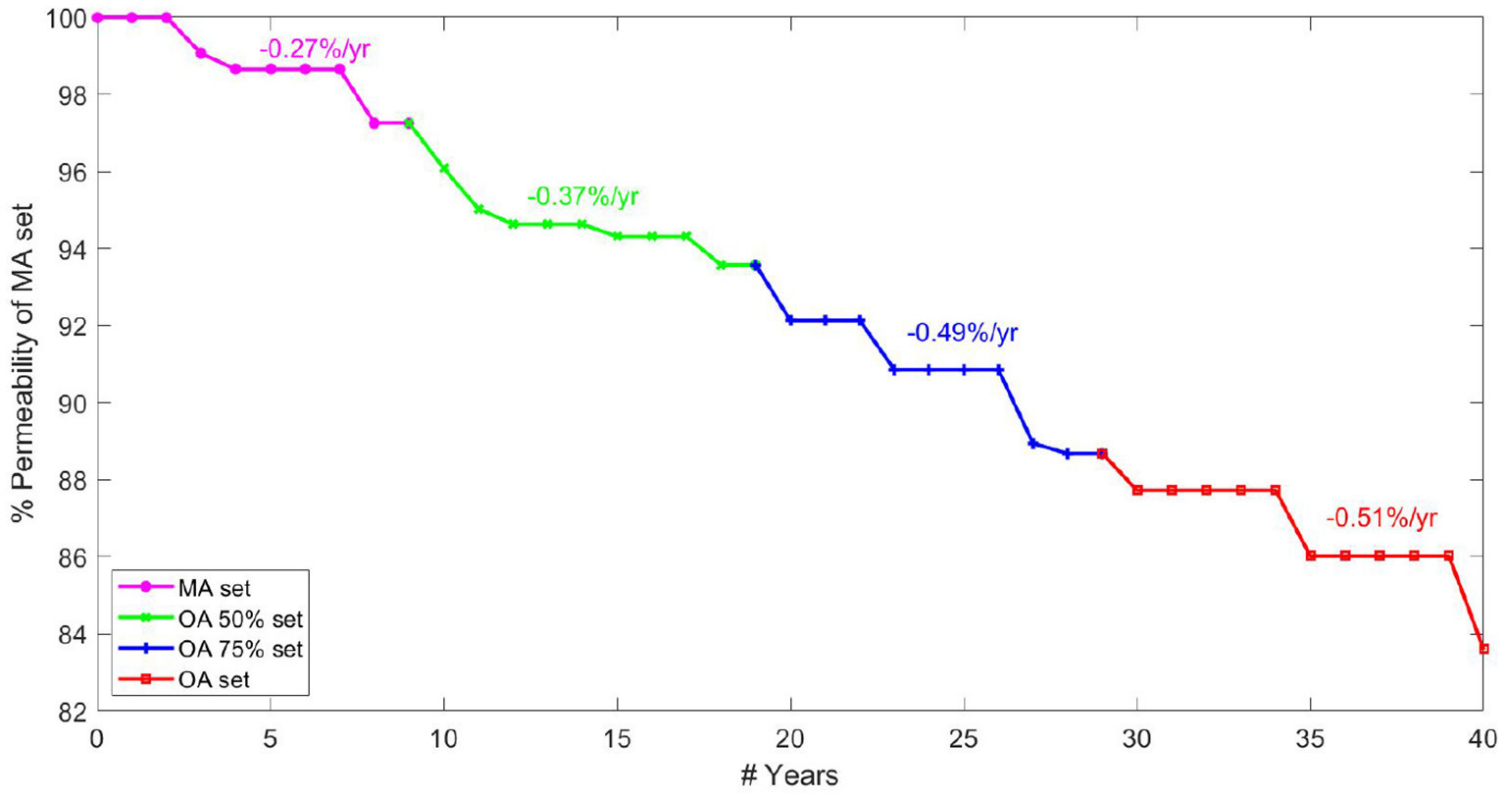
Percentage permeability drop per year in healthy ageing starting from 45 years old ranging up to 85 years old.

The rate of permeability decline at different ages is a key indicator of network robustness with ageing in terms of the damage caused by individual vessel loss and morphometric changes. A continuous gradient for drop in permeability per year allows capillary bed health to be analysed for a healthy individual between middle-age and old-age. Modelling four rates of brain ageing from age 45, data are presented in terms of a percentage permeability drop per year. Note that the percentage change in Figure 8 is calculated relative to the permeability in the MA network before pruning. Plateaus in the figure were due to the fact that a decrease in target CBF did not necessarily correspond to an entire vessel being removed each year (where the decrease in CBF is averaged over all networks in that age bracket).

The graph in Figure 8 is created by combining calculations of percentage permeability drop per vessel and calculations for expected vessel reduction per year, to give a permeability drop per year in the cerebral capillary bed. Analysis of expected vessels lost per year, calculated by matching cerebral blood flow drop in ageing, suggests that fewer vessels are lost each year in old age than in the middle-aged brain. However, the gradient of permeability decline per vessel lost increases with increasing age, showing increased sensitivity of the cerebral permeability to vessels being pruned. This shows lower robustness of the permeability to structural changes in OA.

The results show an increasing rate of permeability reduction per year with age. The rate of decline increases by 35% from MA to OA 50 sets / OA 50 sets to OA 75 sets but only by 5% from OA 75 to OA sets. This contrasts with the expectation that most serious cases of cognitive decline are seen after the age of 75. For example, rates of dementia in the population increase by 0.5% per year up to the age of 75 and by 6–8% per year above that (Checkoway et al., 2011). This potentially indicates that the human brain reaches a tipping point, after which small permeability changes have a much larger impact. Therefore cerebral ageing might be described as a period of microvascular decline before manifestation in actual cognitive function, explaining the timeframe of increased rate of brain deterioration.

Linear deterioration between age sets in discrete time measurements was assumed. Testing this would require more discrete measurements to be taken, but the assumption is frequently made (Ziegler et al., 2012) and agrees with many measurable biomarkers of ageing (Finch and Crimmins, 2016). However, this does not correspond to life expectancy as ageing is a very individual process due to lifestyle and underlying health. The primary value of the work presented here lies in understanding the trend of healthy ageing. There is also potential to look at the relative brain age of individuals, who follow the trend observed offset by a certain number of years. If imaging techniques develop to measure capillary density *in-vivo*, it will be possible to longitudinally study individuals and assess their ageing trajectory. This could potentially allow identification of “unhealthy” ageing before symptoms appear.

### 3.5. Micro-Emboli in the Ageing Cerebral Capillary Bed

**Figure 9:**
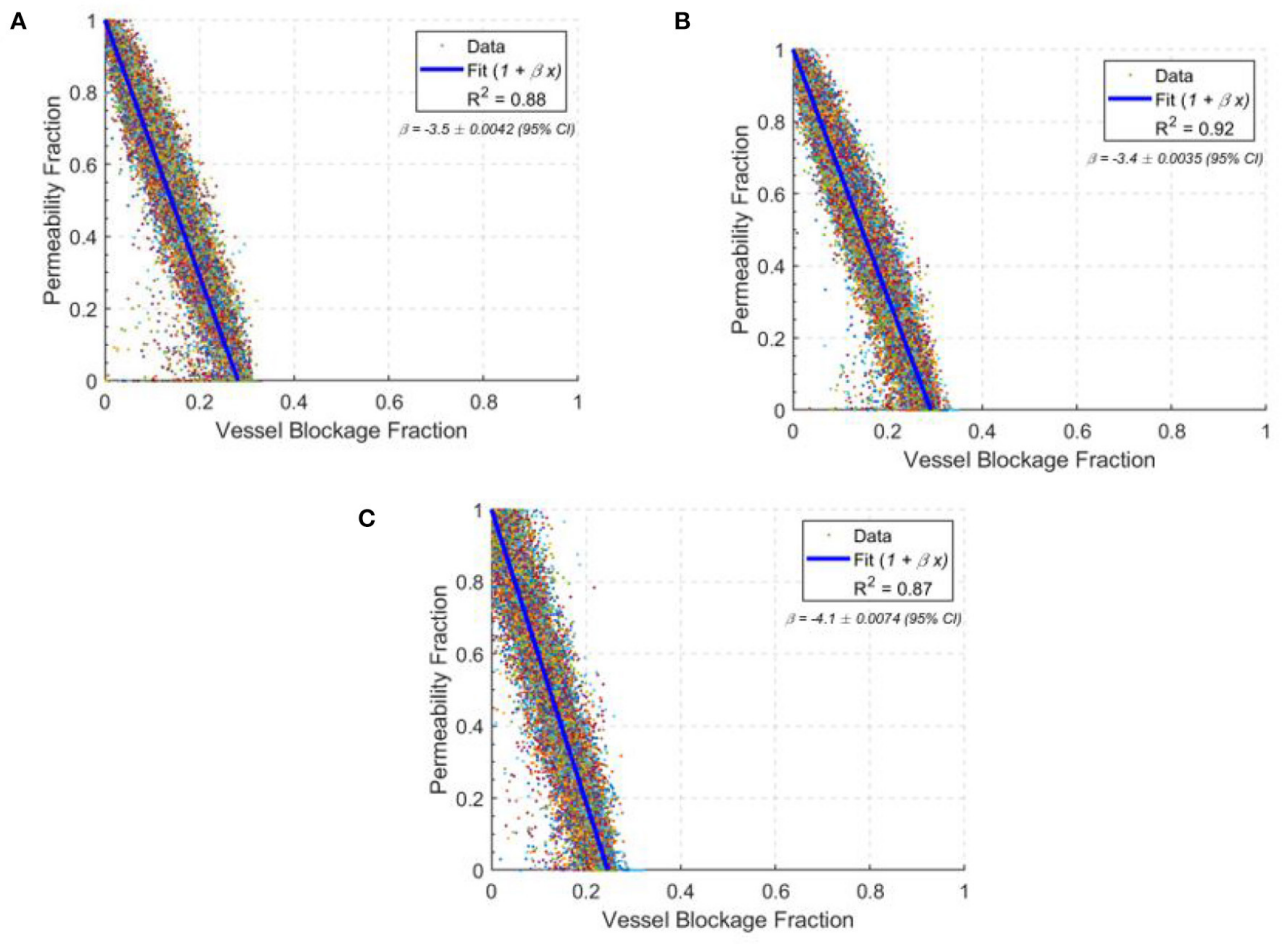
The drops in permeability per vessel blocked in young **(A)**, middle-age **(B)**, and old-age **(C)** for statistically accurate capillary networks. Lines of best fit are in blue with values of the gradient given in the top right corner of each graph. Each data point shows the permeability fraction at any given vessel blockage fraction for a given network, with 166 networks simulated at each age group.

The permeability drop in the middle-aged capillary bed was calculated to be: −8.5 % / % surface area lost, −3.4 % / % vessels blocked and −4.3 % / % volume blocked. This is comparable to the results in section 3.4 for the ageing gradient, and also with synthetic mice models in the literature (Cruz Hernández et al., 2019). Figure 9 shows the scatter distribution of 166 middle-aged and 166 old-age network cubes for the vessel blockage fraction. For each cube, a number of vessels were blocked until the overall permeability reached zero. These were then all plotted to show the reduction rate in permeability from 100 to 0% i.e., each cube has multiple points in Figure 9 demonstrating the impact of successive occlusions on the blood flow.

The permeability drop in the old-age capillary bed was calculated to be: −9.5 % / % surface area lost, −4.1 % / % vessels blocked and −4.9 % / % volume blocked. This shows a faster permeability decline in old-age than in middle-age, which is consistent with the reduction in network robustness described earlier. The changes in rate of permeability decline from middle-age to old age are: +11.2% for surface area lost, +18.5% for vessels blocked and +14.9% for volume blocked. Therefore old-age has a significant effect on the response of the cerebral capillary networks to micro-emboli.

Interestingly, the permeability drop in the young capillary bed was calculated to be: −8.8 % / % surface area lost, −3.5 % / % vessels blocked and −4.3 % / % volume blocked. The changes in rate of permeability decline from the young-age group to the middle-aged group are: +3.5% for surface area lost, +2.9% for vessels blocked and −0.4% for volume blocked. Therefore, the impact of occlusions on young and middle-aged networks are largely similar, but in old-aged networks the impact of an occlusion is much larger.

There is also around twice the variability in the old age group in comparison to the middle-aged group and 20% greater variability in the young age group compared to the middle-aged group, illustrated by the widening 95% confidence intervals. This is consistent with the idea that as the size of the network reduces, specific individual vessels will play more of a critical role in perfusion and if these are randomly blocked, an oversized effect on the permeability and molecular exchange will be seen.

## 4. Discussion

In this paper, a computational model of the ageing brain was developed. Brain microvascular robustness is shown to decrease significantly with age, particularly with regards to permeability. The analysis of continuous ageing indicates that the majority of significant structural changes in the cerebral capillary bed occur before old-age, the expected time frame for cognitive decline. Therefore the model captures early biomarkers in microvascular health leading to deterioration of brain functionality. In addition, testing the robustness of the capillary bed to micro-emboli in ageing confirms increased vulnerability of the old age capillary bed, as well as increased variability between individuals with ageing.

The permeability and molecular exchange distributions for ageing show significant reduction from middle-age to old-age, as well as increased variability in old-age. The findings, especially the drop in permeability, show that healthy ageing will still affect the baseline perfusion of the capillary bed. In future work it will be important to quantify how this extends to the larger vessels, by coupling arteriolar and venular trees to the homogenised cubes in whole brain perfusion models. The deterioration seen in healthy ageing is replicated at extremes in neurodegenerative diseases, and this illustrates the difficulty in identifying how “healthy” the rate of ageing is in the brain.

The continuous gradient of the ageing model proposed here attempts to tackle this problem by proposing a method for comparing discrete longitudinal studies charting the reduction of the permeability in the capillary bed of the brain, a proxy for the health of an individual brain region. A continuous timeline of ageing allows assessment of the relative brain age of an individual, as well as providing a benchmark for a healthy trend in ageing. The rate of ageing is shown to increase from the age of 45. However, the sharpest increase in rate of decline comes before the age of 75, which is considered to be a threshold for dementia onset. This suggests a tipping point of the ageing network, beyond which smaller changes become much more physically significant. This model tracks small-scale deterioration before changes become apparent in brain function. The hypothesis of a critical threshold or tipping point in the cerebral circulation that leads to pathologies characterizing dementia and Alzheimer's has previously been postulated—a critically attained threshold of cerebral hypoperfusion (CATCH) (De la Torre, 2000). The model presented here supports this theory, clearly demonstrating a threshold in the integrity of the cerebral microvascular network after which small vascular changes lead to large changes in the permeability.

The test case of modelling micro-emboli in the cerebral capillary bed clearly shows the increased vulnerability of the brain microvasculature in ageing. There is also increased variability which is consistent with increased variability of permeability and molecular exchange parameters in old age. This is an initial characterisation of the use for an ageing brain model as a tool to simulate important clinical events. Further extensions of this idea, as well as combining the results with macro-scale models, will create models of more complicated ageing scenarios with comorbidities, and potentially even disease states such as Alzheimer's Disease. This will allow a direct comparison between healthy ageing and a patient state to inform better treatment.

Brain integrity and functionality is guaranteed through an energy balance, with a steady supply of oxygen from the microvasculature to the tissue. As the brain ages healthily, despite a reduction in vascular density, tissue cell density appears to remain constant (Thulborn et al., 2016). However, the vascular network is now much more susceptible to minor changes, as demonstrated in this paper using micro-emboli. Therefore, minor changes in the vascular structure can lead to energy imbalance and regional neuronal cell loss over a period of years or decades. Future research will look at adding an oxygen metabolism and tissue cell death model to simulate this behaviour.

It is important to recognise the limitations of assumptions made here. Many of the outputs are driven by the measurements taken in Moeini et al. (2018), a paper with limited comparable literature for corroboration. The parallel assumption of mice and human ageing creates uncertainty, especially due to the regional discrepancies known to exist in the brain. The region of the brain observed in Moeini et al. (2018) has no direct equivalent in humans. It is therefore also different to the brain region upon which the underlying model is based. It should, however, be noted that previous research has demonstrated a topological equivalence in the cerebrovascular capillary networks of mice and humans that reproduces both structure and function of the networks (Smith et al., 2019a). In addition, the data are measured from three differing populations of mice rather than longitudinally so confounding factors between individuals cannot be isolated (Salthouse, 2012). Furthermore, we assumed the length of the capillary vessels does not change with age. This assumption helped simplify the modelling but did not account for previously demonstrated increases in capillary length with age—primarily due to an increase in tortuosity of the vessels (Kalaria and Hase, 2019; Propson et al., 2021). The impact of the increased tortuosity on blood flow and permeability will be 2nd order, however, as radius dominates blood flow dynamics in vessels through Poiseuille's Law. A further limitation of this study is the neglect of phase separation of the red blood cells in the capillary bed. When simulating oxygen transport on the local scale this can have a large effect and should be accounted for, although the impact on permeability will be minor.

There is a lack of clarity over the term “healthy” ageing due to the high frequency of comorbidities in the elderly, such as hypertension or diabetes, as well as the large impact lifestyle, diet and social participation have. These have large effects on ageing and therefore the “healthy” old age modelled here is a relatively rare physiological scenario. The current model architecture cannot capture certain parameters which change with age, such as tortuosity and stiffness. The cerebral capillary bed is also heavily reliant on the upstream properties of the vasculature, as the autoregulatory capacity of the arteries shields / exposes the capillaries themselves. Without quantifiable coupling of the capillary bed to larger vessels, this model must thus remain a standalone tool. Furthermore, the vascular component of ageing is only one facet of an incredibly complex process which depends on genetics, neurotransmitters and hormones, amongst other factors, that determine brain ageing (Peters, 2006). By simply focussing on the vascular component, significant heterogeneity will be missed and needs to be addressed in future work. However, the attempt to standardise “healthy” ageing will provide useful insights in identifying what effects these other factors can produce in the microvasculature.

The next stage is to increase the physiological accuracy of this model. Modelling the interactions between penetrating arterioles and the capillary bed will show how the changing parameters upstream may effect what is observed downstream or vice versa (Adams et al., 2015). In particular, the penetrating arterioles have a large impact on oxygen flux to the tissue and hence would need to be modelled when simulating oxygen transport (Sakadžić et al., 2014). This will allow prediction of global changes from capillary bed development and show how the role of the capillary bed changes with age.

The nature of the model allows separate quantification of the effects that different physiological changes have on ageing. The reduction in vessel density observed in the literature is thought to be due to declining levels of growth factors throughout the body (Ambrose, 2017). Capturing the effect of the subsequent dilation of the remaining vessels on the network flow and permeability illustrates the excess capacity of the cerebral microvasculature in a healthy adult brain. Understanding the reduction of this in ageing is an interesting area for further exploration, especially in the context of common comorbidities in old age. In a healthy brain, there is a buffer as the vessels can dilate, at least up to the radius seen in old age. In old age, it is expected this buffer reduces as there is less ability of the vessels to dilate beyond the old age radius, and therefore the vessels are nearer functional capacity.

One of the major outputs of this paper is the healthy ageing gradient. This has the capability to use potentially measurable parameters (haematocrit, CBF, radius) to create a clinically useful tool. Ability to measure these parameters *in-vivo* in humans is improving rapidly. This, combined with the rise of personalised medicine, will see more regular scans and tests to enable tracking of “healthy” ageing based on a more extensive medical history. A tool to map “relative brain age” of an individual would summarise whether there was unexpected deterioration. This could be used to inform suggestions for lifestyle changes, or further research into factors that affect ageing in the population. An equivalent gradient for Alzheimer's Disease could highlight differences over time in the health of the capillary bed, potentially before they manifested in brain function. After comparison, a prediction method of future brain health based on lifestyle and physiological measurements could be built from this information. It should be noted that currently this model does not account for sex-based differences or comorbidities. However, the model inputs are flexible enough such that we should be able to account for sex-based differences as well as comorbidities such as diabetes or hypertension—as long as the data is available.

Brain computer models allow *in-silico* estimation of blood flow properties and insight into clinically unmeasurable parameters. However, they are currently sparsely used in clinical practice. A working computer model of the brain microvasculature in ageing would allow earlier identification of “at-risk” groups for cerebrovascular degeneration, to inform lifestyle changes and medical intervention. This could be achieved through comparisons of “healthy” and “unhealthy” ageing based on more standardised definitions. Physiological parameters measurable *in-vivo* such as cerebral flood flow can be fed into the model to determine patient risk. Deeper understanding of inhibited localised flow patterns and potential new ideas to target biomarkers of disease will maximise cost effectiveness of treatment and patient outcomes.

## Data Availability Statement

The original contributions presented in the study are included in the article/supplementary material, further inquiries can be directed to the corresponding author/s.

## Author Contributions

BG developed the ageing brain model, conducted the analysis, produced the figures, and wrote the paper. WKE-B and SJP designed and supervised the research. All authors contributed to the revision of the paper.

## Conflict of Interest

The authors declare that the research was conducted in the absence of any commercial or financial relationships that could be construed as a potential conflict of interest.

## Publisher's Note

All claims expressed in this article are solely those of the authors and do not necessarily represent those of their affiliated organizations, or those of the publisher, the editors and the reviewers. Any product that may be evaluated in this article, or claim that may be made by its manufacturer, is not guaranteed or endorsed by the publisher.
